# Crisis biopolíticas actuales: una mirada crítica a las nuevas eugenesias en América Latina

**DOI:** 10.1590/S0104-59702026000100040

**Published:** 2026-10-12

**Authors:** Francisco Mena Mena

**Affiliations:** i Académico, Universidad Católica de la Santísima Concepción. Concepción – Chile. francisco.mena@ucsc.cl

**Figure:**
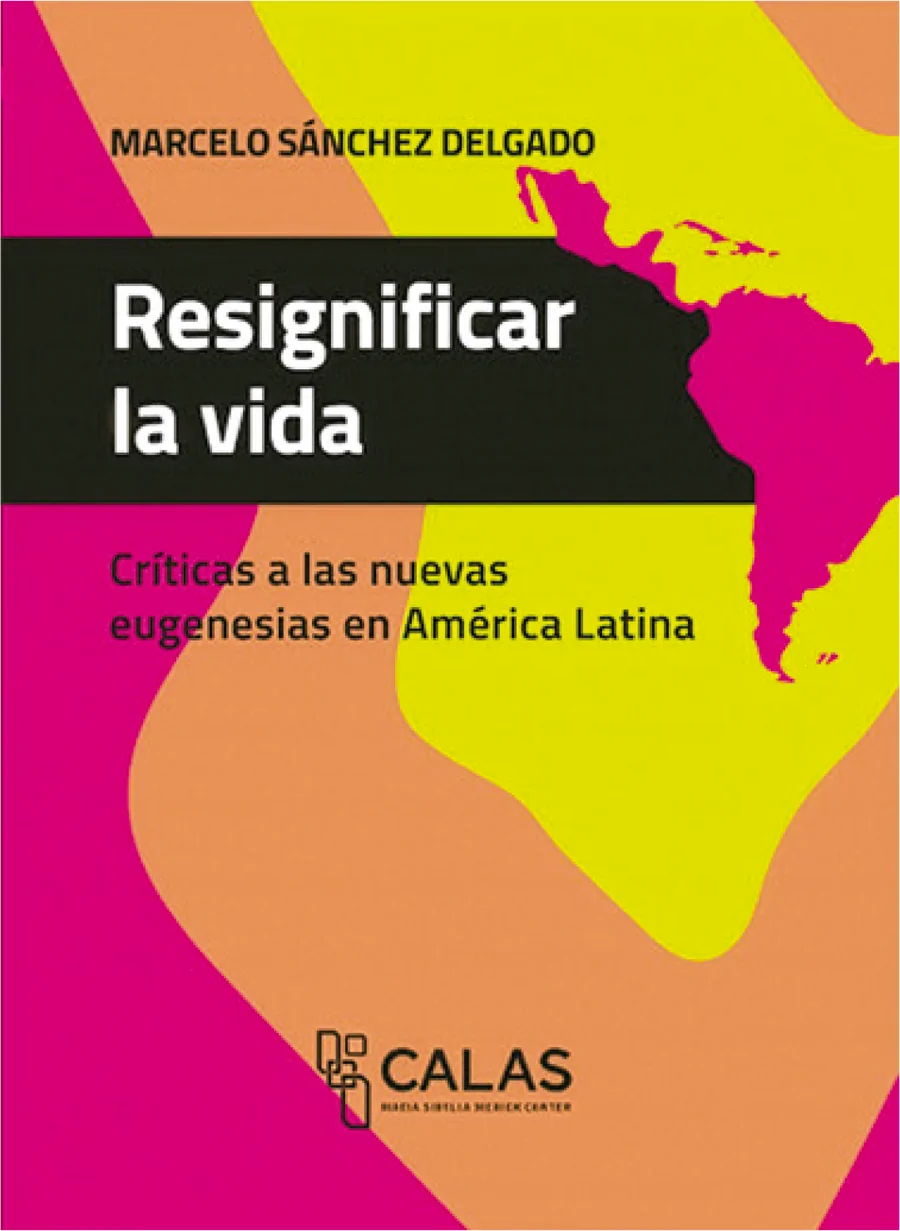

¿Es posible hablar de nuevas eugenesias en el siglo XXI latinoamericano? ¿Continúan vigentes, bajo otras formas, las ideas de perfeccionamiento humano y racial surgidas en el siglo XIX? En *Resignificar la vida*, el historiador Marcelo Sánchez Delgado propone una lectura crítica sobre la operatividad contemporánea de la eugenesia en América Latina, alejándose de los enfoques tradicionales y enfocándo en sus reconfiguraciones tecnocientíficas, políticas y culturales. Su ensayo, lejos de ser una cronología exhaustiva o una revisión historiográfica al uso, busca indagar cómo los discursos biomédicos y los dispositivos de control biopolítico siguen configurando proyectos de vida y muerte en la región.

La obra se inscribe en el contexto de aceleración de áreas como la biotecnología, la genética y la inteligencia artificial, donde los paradigmas contemporáneos de gestión corporal y poblacional no responden necesariamente a la clásica relación Estado-nación-raza, sino a estructuras neoliberales, privadas e individualistas (Habermas, 2002) que operan bajo criterios humanitarios, terapéuticos y científicos. Sánchez Delgado (2024, p.19) sugiere que esta aparente neutralidad encubre la vigencia de idearios eugenésicos que continúan gestionando lo humano desde la racionalidad tecnocientífica y la promesa de un futuro perfeccionado, por lo que propone “indagar en las formas adquiridas por la eugenesia en los campos científicos contemporáneos y en el discurso-práctica del poder sobre los actuales problemas sociales latinoamericanos”.

De esta manera, se plantea la necesidad de posicionar a América Latina en los debates globales sobre las nuevas eugenesias, construyendo un relato comparativo y multitemporal que conecta experiencias de países como Brasil, Chile, Argentina, México, Cuba y Guatemala. Aún así, su autor reconoce la crucial dificultad de aunar una sistematización continental, optando por ejemplos significativos que permiten identificar la plasticidad y vigencia de prácticas eugenésicas en escenarios diversos. En consecuencia, se observa un alejamiento de la historiografía monolítica, favoreciendo un abordaje interdisciplinario que permite pensar críticamente las crisis actuales desde su articulación con saberes biomédicos y discursos sobre el futuro humano.

En cinco capítulos, además de un manifiesto final, Sánchez revisa casos concretos de reactivación eugenésica y sus vínculos con fenómenos como el crimen, la migración, la natalidad y el transhumanismo. En el primer capítulo, Sánchez analiza la megacárcel salvadoreña “Cecot” como dispositivo de control biopolítico. El autor vincula esta estrategia represiva con la tradición eugenésica de selección y exclusión, remitiendo a prácticas como la identificación por características físicas y pobreza, retomando la discusión sobre el uso de marcadores biológicos como la huella dactilar, a pesar de su notoria connotación estigmatizadora.

El segundo capítulo, se enfoca en la crisis de natalidad en América Latina y la reactivación de debates sobre reproducción y género. Partiendo del reportaje “Jalisco se queda sin niños”, de 2023, se discute cómo la caída demográfica se vincula con transformaciones en el rol de la mujer y la reconfiguración del binomio madre-hijo. Sánchez señala que, aunque la esterilización parece haber perdido su impulso estatal, sigue presente en sistemas de salud públicos y privados, funcionando como herramienta de control y segregación bajo criterios de género y herencia degenerada. Plantea, además, que el futuro demográfico será objeto de disputa política y biopolítica.

A continuación, en el tercer capítulo, el autor aborda la inteligencia como criterio clásico de clasificación eugénica, revisitando sus vínculos con los discursos de superación humana en contextos de aceleración tecnológica. Apoyándose en los análisis de intelectuales como Timnit Gebru y Émile Torres, críticos vigentes de las “nuevas eugenesias” o de “libre mercado”, siguiendo las clasificaciones de Michael Sandel (2007), se discuten los riesgos ético-políticos de doctrinas transhumanistas que promueven el abandono de la naturaleza humana como camino a la perfección. De esta manera, se propone una genealogía crítica del acrónimo Tescreal, que agrupa corrientes como el transhumanismo, el singularismo y el largoplacismo, articulando sus vínculos con los discursos eugenésicos del siglo XX.

El cuarto capítulo, analiza la gestión migratoria en clave eugenésica, comparando políticas actuales con normativas de exclusión racial y biológica del pasado. Acá, el historiador reflexiona sobre los discursos que buscan controlar la entrada de cuerpos migrantes en función de su potencial reproductivo y social, retomando la política argentina de “gobernar es poblar” y su correlato en Brasil y Chile. La tensión entre inclusión y exclusión aparece como eje clave para entender cómo se proyecta la noción de peligrosidad en los flujos migratorios contemporáneos.

Finalmente, el quinto capítulo, parte de la pandemia de covid-19 para discutir cómo persisten las lógicas de selección de vida y muerte. Basándose en las cifras de mortalidad de grupos marginados en EEUU y Brasil, se evidencia la continuidad en las lógicas de la “lucha por la existencia”, y su conexión histórica con otros contextos de administración sanitaria, como la exclusión de enfermos hacia Isla de Pascua en Chile. La obra plantea que las nuevas eugenesias, impulsadas por corrientes como el transhumanismo, están definiendo los caminos hacia una reorganización del ser humano, lo animal y lo natural, bajo la promesa de un futuro más “digno”.

En suma, *Resignificar la vida* se erige como un esfuerzo crítico por desentrañar las continuidades y transformaciones de la eugenesia en América Latina, estableciendo puentes entre las prácticas del siglo XX y los dispositivos biopolíticos actuales, evidenciando cómo estas ideologías siguen operando en un contexto neoliberal bajo discursos de bienestar y calidad de vida. El manifiesto final, una apuesta por una “biopolítica plebeya” frente a la lógica de élite en las crisis biopolíticas actuales, reafirma el llamado político a repensar el futuro humano desde un enfoque inclusivo, situado y crítico.

## Data Availability

No están en repositorio.
